# Size Matters: Predicting Surgical Site Infection After Whole Breast Radiotherapy in the Era of Hypofractionation

**DOI:** 10.3390/jcm14010184

**Published:** 2024-12-31

**Authors:** Sea-Won Lee, Yeong Ji Kim, Jae Won Song, Mina Yu, Jiyoung Rhu, Pill Sun Paik, Yong Hyuk Kim, Yun Hee Lee

**Affiliations:** 1 Department of Radiation Oncology, Eunpyeong St. Mary’s Hospital, College of Medicine, The Catholic University of Korea, Seoul 03312, Republic of Korea; lords_seawon@hotmail.com; 2 Department of Radiation Oncology, Bucheon St. Mary’s Hospital, College of Medicine, The Catholic University of Korea, Seoul 14647, Republic of Korea; amything@naver.com (Y.J.K.); song97zzang@naver.com (J.W.S.); persiancat16@catholic.ac.kr (M.Y.); 3 Department of Surgery, Bucheon St. Mary’s Hospital, College of Medicine, The Catholic University of Korea, Seoul 14647, Republic of Korea; jyses82@naver.com (J.R.); pillsun@gmail.com (P.S.P.); 4 Department of Pediatrics, Division of Endocrinology, Wonju Severance Christian Hospital, Wonju 26426, Republic of Korea; sumergo@yonsei.ac.kr

**Keywords:** surgical site infection, breast cancer, radiotherapy, hypofractionation

## Abstract

**Objectives**: Few studies have analyzed surgical site infections associated with hypofractionated RT. The purpose of this study was to identify risk factors for surgical site infections with a particular focus on volumetric parameters that reflect the size of the volumes treated, including tumors, surgical cavities, and breasts. **Methods**: A total of 145 early breast cancer patients who were surgically staged 0—II undergoing hypofractionated RT on the whole breast were retrospectively reviewed. Tumor size (cm) was measured from surgical pathology. Surgical cavity volume (cc) and breast volume (cc) were calculated by segmenting each axial slice of simulation CT. The cavity-to-breast ratio (%) was calculated as surgical cavity volume/breast volume × 100. **Results**: The incidence of surgical site infection was 4.8% at a median of 6.3 months after the completion of RT. In univariate analysis, tumor size (OR 2.01, *p* = 0.025), surgical cavity volume (OR 1.03, *p* = 0.013), cavity-to-breast ratio (OR 1.29, *p* = 0.005), and BMI (OR 1.23, *p* = 0.014) were significantly associated with surgical site infection. In multivariate analysis, the cavity-to-breast ratio (OR 1.24, *p* = 0.039) remained significantly associated with surgical site infection. **Conclusions**: This study highlights the importance of volumetric parameters, specifically the cavity-to-breast ratio, as significant predictors of surgical site infection in a pure cohort of early breast cancer patients undergoing breast-conserving surgery and hypofractionated RT. Tailored approaches, including the use of prophylactic antibiotics, prophylactic aspiration, and close follow-up, may reduce the morbidity associated with surgical site infection and prevent the potential compromise of tumor outcomes.

## 1. Introduction

The COVID-19 pandemic influenced cancer care globally, prompting the widespread adoption of hypofractionated radiotherapy protocols to reduce hospital visits and minimize exposure risks while ensuring the continuity of treatment. This was also true for early breast cancer, as moderately hypofractionated whole breast irradiation had already been gaining recognition as the standard of care for adjuvant radiation therapy (RT) [1], supported by compelling evidence from randomized controlled trials demonstrating comparable outcomes with conventional RT in terms of recurrence and toxicity [2,3,4,5,6,7]. While such trials have found reductions in dermatitis, edema, and pain associated with the breast, the risk factors for surgical site infections following hypofractionated RT remain elusive.

The disruption of the breast lymphatic system from breast and axillary surgery, when compounded by RT-induced tissue changes, may exacerbate lymphatic flow obstruction. The presence of subcutaneous fluid collection poses a potential risk of becoming a reservoir for infection [8]. Surgical site infections can lead to patient morbidity, requiring additional treatments and prolonged hospital stays, which can undermine patient well-being and increase healthcare costs [9]. Surgical site infections may act as a barrier to further treatment, potentially compromising tumor outcomes [10]. A comprehensive understanding of the risk factors for surgical site infections following breast conserving surgery (BCS) and hypofractionated RT has the potential to not only improve the overall quality of life for patients but also increase the chances of a better oncologic outcome.

The existing literature has identified patient-specific risk factors for surgical site infections, including age and comorbidity, alongside perioperative factors such as surgical technique and postoperative wound care [11]. Modern RT techniques enable the precise volumetric assessment of treated regions, including surgical cavities and whole breasts. However, the implications of these volumetric parameters—particularly in the context of hypofractionated RT—are still underexplored, despite their potential impact on postoperative healing, tissue response to RT, and infection susceptibility [12].

This study aims to evaluate surgical site infections after hypofractionated RT by identifying potential contributing factors with a specific focus on volumetric parameters. Our goal is to improve patient selection for early intervention, allowing tailored approaches to minimize the risk of surgical site infections following contemporary RT.

## 2. Materials and Methods

### 2.1. Patient Accrual

This study retrospectively examined consecutive patients referred for postoperative whole breast irradiation who were surgically staged as 0—II by the 8th edition of the AJCC staging system [13] from January 2019 to July 2021. Inclusion criteria were as follows: patients with (1) histologically proven breast cancer; (2) primarily treated with BCS; (3) pathologic stage 0—II; (4) completed initially planned whole breast RT; and (5) RT fraction size of 2.5–3.5 Gy (moderate hypofractionation). Exclusion criteria were as follows: (1) previous history of radiotherapy to the breast, thorax, or head and neck; (2) received neoadjuvant chemotherapy; (3) documented distant metastasis from diagnosis to the time of radiotherapy; and (4) conventional fraction size of 1.8–2 Gy. The entire process of the current study (protocol code: HC21RISI0123) was confirmed by the institutional review board, which acquired full accreditation by the Association for the Accreditation of Human Research Protection Programs (AAHRPP).

### 2.2. Data Collection

Baseline patient characteristics previously reported as potential risk factors for surgical site infection such as age, comorbidities, height, weight, body mass index (BMI), smoking history, and alcohol intake were collected from electronic medical records (EMRs). Information regarding the types of breast and axillary surgery was obtained from operation records. Tumor characteristics, including tumor size, pT stage, pN stage, and molecular status, such as estrogen receptor (ER), progesterone receptor (PR), and human epidermal growth receptor 2 (Her2), were collected from the surgical pathology reports.

### 2.3. Treatment

The treatment approach for each patient diagnosed with breast cancer at the institution is determined through the consensus of a multidisciplinary panel. The primary framework for determining the mainstay of treatment for early breast cancer patients is guided by the NCCN guidelines [14]. While clipping is considered the standard practice for tumor bed localization, our institution primarily employed the open cavity technique in BCS to ensure optimal oncologic outcomes and reduce the need for re-excisions. Tumor localization was achieved through simulation CT imaging of the surgical cavity combined with preoperative imaging, which served as the primary method for defining the tumor bed area. Level I oncoplastic surgery was the primary approach for oncoplastic surgical techniques. In cases where level II oncoplastic surgery was considered feasible, patients were informed of the options available, and level II surgery was performed when appropriate. The decision regarding the surgical approach for axillary management was made by a surgical oncologist. Adjuvant chemotherapy, hormonal therapy, and targeted therapy were selected at the discretion of the treating physician according to molecular characteristics. RT field and dose fractionation were specified by the radiation oncologists. For patients with minimal risk of axillary spread, RT was limited to the residual breast parenchyma. In cases with nodal disease requiring axillary dissection, the axilla was actively incorporated into the RT field. A sequential boost to the tumor bed was delivered, with a dosage of 10–12.5 Gy in 4–5 fractions. The treatment sequence was structured with surgery as the first step, followed by adjuvant systemic therapy if indicated, and then radiotherapy and hormonal therapy with or without the continuation of targeted therapy.

### 2.4. Volumetric Parameters as Potential Risk Factors for Surgical Site Infection

**Figure 1:**
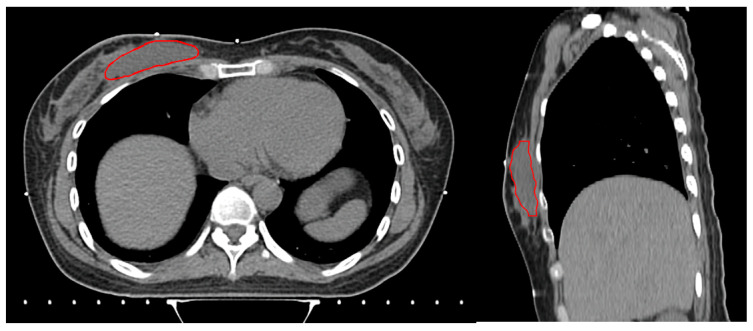
Segmentation of surgical cavity shown in axial (**left**) and sagittal (**right**) views of simulation CT.

The dimensions of the surgical specimen, including width (cm), depth (cm), and height (cm), as well as the width and depth (cm) of the excised skin, were obtained from the gross pathology report. Skin depth and width were measured from the surgical pathology specimen. Skin depth was defined as the vertical thickness from the epidermis to the dermis and skin width as the lateral dimension of the excised skin. Measurements were taken from a representative area of the specimen using standardized calipers. The specimen volume (cc) was calculated by multiplying the width, depth, and height of the surgical specimen. Breast volume (cc) and surgical cavity (cc) were calculated by segmenting each axial slice of simulation computed tomography (CT) according to the RTOG guidelines [15] using MIM (MIM Software Inc., Cleveland, OH, USA). Surgical cavity was defined as a localized fluid attenuation, separate from the surrounding breast parenchyma on a CT image (Figure 1).

**Figure 2:**
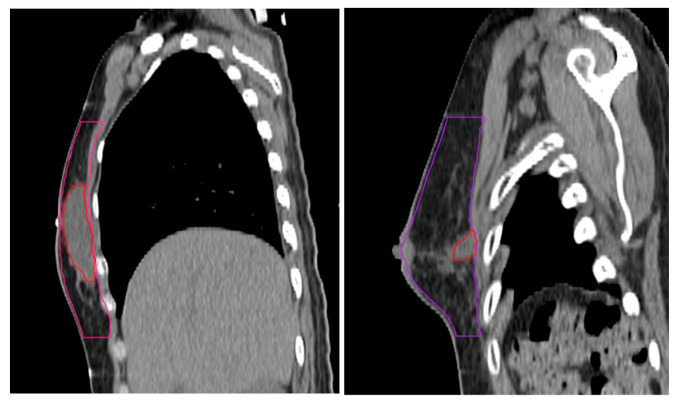
Segmentation of whole breast and surgical cavity with large (**left**) and small (**right**) cavity-to-breast ratios.

A single radiation oncologist specializing in breast cancer uniformly segmented the volumes (YHL). The cavity-to-breast ratio (%) was calculated as follows (Figure 2):

Cavity-to-breast ratio (%) = (Surgical cavity volume (cc))/(Breast volume (cc)) × 100

### 2.5. Toxicity Assessment

Surgical site infection was defined as any subjective sign of inflammation, such as erythema, warmth, swelling, or pain, combined with at least one of the objective signs of infection, such as purulent discharge, a positive response to infection treatment (such as antibiotics, incision, drainage, or wound revision), or documented microbial positivity. Importantly, the affected area was mainly confined to the surgical bed. RT toxicity was monitored weekly from the initiation to completion of whole breast irradiation. Common toxicities associated with RT, including radiation dermatitis, breast pain, breast edema, and arm edema, were observed during weekly clinical examinations. Toxicities were assessed and graded according to the CTCAE v5.0. [16]. Acute toxicity after RT was defined as up to 3 months after the completion of RT. Any adverse effect that occurred more than 3 months after RT was considered to be late or delayed toxicity. Routine follow-up examinations after RT were performed every 6 months up to 5 years. Imaging studies such as ultrasonography, mammograms, CT, magnetic resonance imaging (MRI), bone scans, and blood tests were performed accordingly.

### 2.6. Statistical Analysis

Descriptive statistics were used to identify potential risk factors for surgical site infections, including patient, tumor, and treatment characteristics, as well as volumetric parameters. The values were presented as the median and range. The primary endpoint was surgical site infection. Secondary endpoints were any RT-related toxicities. The association between risk factors and surgical site infections were analyzed using a logistic regression analysis, presented with the odds ratio (OR) and 95% confidence interval (CI). The factors were analyzed as continuous variables. The cut-off value was obtained using the receiver operating characteristic (ROC) curve analysis. Statistical significance was defined at *p* < 0.05. All statistical analyses were performed using R software (R Core Team 2023).

## 3. Results

### 3.1. Patient, Tumor, and Treatment Characteristics

A total of 145 patients with stage 0-II early breast cancer treated primarily with BCS followed by whole breast RT who met the inclusion criteria were analyzed (Table 1).

The cohort had a median age of 54 (range: 26–82). The median BMI was 24.2 (range: 17.7–38.3). The majority of the patients (89.7%) had no smoking history. A total of 11.7% were diagnosed with DM and 29.7% with HTN. All patients underwent level I oncoplastic surgery, except for one patient who received partial breast reconstruction with muscle-sparing latissimus dorsi flap coverage. Axillary surgery was performed in the order of SLNB (86.2%), ALND (6.2%), and none (7.6%). The majority of the patients were staged at pT1 (55.9%), followed by pT2 (22.1%) and pTis (21.4%). Most of the patients were diagnosed as node-negative (85.5%) with N1a in 10 patients (6.9%). Half of the patients underwent adjuvant chemotherapy (50.3%). Tamoxifen (40%) and aromatase inhibitor (45.5%) were administered in line with hormone positivity. Similarly, 9.7% of patients received adjuvant targeted therapy based on their Her2 status. For the moderate hypofractionation regimen, the radiation doses of 4005 cGy in 15 fractions (60.7%) and 4256 cGy in 16 fractions (39.3%) were used. In terms of the RT field, the majority were the residual breast parenchyma (N = 135), and in the cases of nodal disease, the axillary nodal stations were included (N = 10). Additionally, a boost to the tumor bed was delivered to the majority of the patients (91%). The median interval between BCS and the initiation of RT was 104 days (range: 20–219). In patients who underwent chemotherapy, the median interval between BCS and RT was 121 days (range: 96–129 days). For patients who initiated RT immediately after surgery, the median interval was 35 days (range: 20–62 days).

### 3.2. Hypofractionated RT Outcome and Toxicity

No recurrence was observed at a median follow-up period of 33 months (range: 4.5–51.8). There was no grade 3 or higher acute toxicity related to RT observed (Table 2).

A total of 4.8% (N = 7) of patients experienced grade 2 acute toxicity after RT. The RT toxicity profile included radiation dermatitis (N = 3), breast pain (N = 3), and breast edema (N = 1). The majority of the patients (98.6%) had grade 1 dermatitis, all of which resolved within 3 months. Approximately a third of patients experienced breast pain (25.5%) and breast edema (37.9%), predominantly at the grade 1 level. There was one patient with grade 1 arm edema, although it was difficult to attribute this solely to RT since she had undergone axillary dissection and multiple cycles of adjuvant chemotherapy. No late RT toxicity was observed.

### 3.3. Surgical Site Infection After Hypofractionated RT

There were seven (4.8%) cases of surgical site infection observed after the completion of RT (Table 2). Among these cases, two patients were classified as grade 1, with symptoms that were successfully managed with conservative measures. A total of five patients required antibiotic treatment. Of these, two patients had grade 3 infections requiring hospitalization and IV antibiotics. One patient was treated with IV cefazolin for 14 days, while the other was treated with IV cefazolin for 3 days and then switched to oral cefaclor for another 3 days. The remaining three patients were treated on an outpatient basis with oral cefixime for 7 days. Two of these patients had grade 3 infections requiring surgical intervention. One patient with a grade 2 infection was successfully treated with oral antibiotics alone. The four patients who had grade 3 infections requiring surgical intervention underwent multiple aspirations (n = 1), incision and drainage (n = 1), irrigation (n = 1), and wound revision (n = 1). Surgical site infections were observed at a median of 6.3 months (range: 3.0–17.5 months) after the completion of RT.

### 3.4. Volumetric Parameters as Risk Factors for Surgical Site Infection

The volumetric parameters as potential risk factors for surgical site infection are shown in Table 3.

The median tumor size was 1.4 cm (range: 0.1–5.0). The median width, depth, and height of the surgical specimens were 7.7 cm, 6.0 cm, and 3.0 cm, respectively. The median specimen volume was 144 cc with a wide range from 9.2 cc to 1417.5 cc. The median width and depth of skin removed were 6.0 cm and 1.7 cm, respectively. In addition, the median breast volume at the beginning of RT was 680.9 cc (range: 191.9–1485.8). The median surgical volume at the time of RT was 13.1 cc (range: 0–216). The median cavity-to-breast ratio was 1.78% (range: 0–16.8).

### 3.5. Risk Factors for Surgical Site Infection

Table 4 presents the logistic regression analyses of risk factors for surgical site infection. These analyses were performed to compare patients with and without surgical site infections and identify significant predictors of infection risk. After comparisons were made based on all parameters from Table 1, Table 2 and Table 3, only the parameters with *p* < 0.10 were included in the univariate analysis of Table 4.

In univariate analysis, tumor size (OR 2.01, *p* = 0.025), surgical cavity volume (OR 1.03, *p* = 0.013), cavity-to-breast ratio (OR 1.29, *p* = 0.005), and BMI (OR 1.23, *p* = 0.014) were significantly associated with surgical site infection. Specimen volume, breast volume, and the moderate hypofractionated RT regimen showed trends toward an association with surgical site infection but did not reach statistical significance. When the association between surgical site infections and grade 2 acute toxicities was analyzed, there was no statistically significant association observed (*p* = 0.996). Because none of the previously reported patient- or treatment-related risk factors for surgical site infection, such as smoking history, DM, HTN, adjuvant chemotherapy, adjuvant hormonal therapy, and adjuvant targeted therapy, showed a significant association with surgical site infection, only factors with *p* < 0.10 are shown in Table 4.

Multivariate analysis was performed for factors significant at *p* < 0.05 in the univariate analysis. Because the cavity-to-breast ratio was calculated directly from the surgical cavity volume (*p* = 0.013) and breast volume (*p* = 0.073), only the factor with the highest significance in the univariate analysis was included in the multivariate analysis to avoid a confounding effect, which was the cavity-to-breast ratio (*p* = 0.005). As a result, the cavity-to-breast ratio (OR 1.24, *p* = 0.039) remained significantly associated with surgical site infection, while BMI (OR 1.18, *p* = 0.071) was marginally associated without statistical significance. The cut-off value of the cavity-to-breast ratio associated with surgical site infection was 4.84% with an area under curve (AUC) of 0.761.

## 4. Discussion

Although surgical site infections following breast-conserving therapy are rare, their occurrence can lead to significant morbidity. The reported rate of surgical site infections after BCS has been reported to be as high as 10% [17]. The incidence of surgical site infections in our cohort was less than 5%. However, it is noteworthy that half of the patients with surgical site infections required hospitalization and surgical management, and more interestingly, they all presented with seromas. Another intriguing observation is that all cases of surgical site infection had a delayed onset, manifesting at least 3 months after the completion of RT. In this study, the most significant factor related to surgical site infections was a volumetric parameter, the cavity-to-breast ratio. Therefore, if patients at higher risk of surgical site infections could be predicted using volumetric parameters such as the surgical cavity volume measured in the pre-RT breast, prophylactic care such as more rigorous follow-up may help reduce the development of delayed surgical site infections.

Since the release of the results from the landmark trials of moderately hypofractionated RT, including the Canadian trial and the UK START trials, it has emerged as the standard regimen for adjuvant RT after BCS [2,3,4,5,6,7]. Several other studies have consistently reported that hypofractionated RT is comparable to conventionally fractionated RT in terms of local recurrence and normal tissue toxicity [18]. However, there is limited research analyzing surgical site infection after hypofractionated RT, especially with regard to delayed surgical site infections. Shaitelman et al. reported the acute toxicities of moderately hypofractionated whole breast RT compared to conventionally fractionated RT [19]. Their findings revealed that hypofractionated RT resulted in less acute toxicities, with wound infection reported in only 1%. Previous reports have reported the incidence of seroma, which they considered to be distinct from wound infection, to be approximately 20%, which were described as “clinically significant”, indicating obvious discomfort or the need to aspirate, drain, or revise the wound [20]. On the contrary, we included all patients with seroma as a surgical site infection, still with an incidence of less than 5%. Of these, only one patient required wound revision, and the others were successfully treated with nonoperative management.

Another previous report suggested that a breast specimen weight > 100 g may be a risk factor for surgical site infection [21]. However, it is important to note that this association was not statistically significant in the report. In contrast to specimen weight, our study focused more on the volume of the surgical cavity and its relationship to breast volume. In this study, the exact volumes of the surgical cavity and the breast were obtained from the reconstructed CT images. There was another study similar to ours that showed a significant association between seroma and delayed breast cellulitis [8]. However, it lacked volumetric assessment, and while we analyzed RT-naive surgical cavity volume, they analyzed seroma, which is already a step closer to the development of surgical site infections by natural history. The definition of “delayed” was also different, referring to 3 weeks post-RT, which would still be acute by our definition, separating acute from delayed by 3 months. Nevertheless, it demonstrated the importance of intramammary fluid collection in the development of breast inflammation, which is consistent with the results of the meta-analysis of risk factors for surgical site infection after breast surgery, which highlighted seroma as a warning symptom and urged that special attention be paid to taking measures to prevent surgical site infection [11]. In light of the results of our study, the prevention of surgical site infection can be advanced prior to the initiation of RT with the surgical cavity volume measured on pre-RT imaging. In the meta-analysis, gentle tissue handling, adequate hemostasis, and tension-free incision closure were suggested as possible surgical measures. The effects of specifics with regards to drainage and its duration on the incidence of seroma are still controversial [20]. In the context of prophylactic antibiotics, a retrospective study reported a significant reduction in surgical site infections with the administration of antibiotics [22]. However, the benefit was limited to patients identified as being at high risk for surgical site infections. Consequently, it should be considered on an individual basis rather than as a routine practice. In addition, prophylactic aspiration can be considered in large seromas or in cases of a high cavity-to-breast ratio before the initiation of RT.

The analysis of risk factors for surgical site infection in this pure cohort of early breast cancer patients treated upfront with BCS followed by RT underscores the importance of considering volumetric parameters in treatment planning. The cavity-to-breast ratio, while uncontrollable, provides an objective metric for early risk stratification, enabling clinicians to identify high-risk patients who may benefit from tailored strategies. These strategies include closer follow-up, extending antibiotic prophylaxis with oral antibiotics after a preoperative single IV dose, or prophylactic aspiration. Furthermore, daily cone-beam CT during RT offers a non-invasive method for monitoring surgical cavity dynamics without additional radiation exposure. For instance, an observed increase in cavity size could prompt earlier interventions, such as prophylactic aspiration, potentially reducing infection risks and improving patient outcomes. To the best of our knowledge, this is the first study on surgical site infection with a volumetric approach in early breast cancer patients treated with hypofractionated RT.

One possible hypothesis is that volumetric parameters may have a greater impact on delayed surgical site infections than previously recognized biological and physiological risk factors, such as underlying comorbidities. In our cohort, none of the previously reported patient-related risk factors, such as age and comorbidity, exhibited a significant association with surgical site infection, except for tumor size and BMI in the univariate analysis. Tumor size is a volumetric parameter, and BMI is an underlying patient-related biophysiological factor. DM and HTN are known to alter tissue microenvironments, promoting inflammation and delayed healing, and are thus more likely to be associated with acute surgical site infections [23,24]. Surgical cavities often serve as the target volume for an additional boost during adjuvant whole breast RT. Delivering a higher dose of radiation to the surgical cavity which is already damaged by tumor resection may increase the risk of significant microvascular injury. The postsurgical disruption of lymphatics, followed by the healing process of the tissue microenvironment and fibrotic changes in the microvasculature, occurs over a prolonged period of time [25], which may explain the delayed onset of surgical site infection. Impaired fluid circulation and retention within the tissue microenvironment can increase the risk of infection by serving as a reservoir for pathogens [8] Thus, delayed surgical site infections may be attributable to structural changes in the tissue microenvironment rather than the immunosuppressive and inflammatory susceptibilities associated with biophysiological risk factors such as DM and HTN reported in previous studies [12]. Furthermore, obesity, as reflected by a high BMI, is recognized as a risk factor for structural changes in tissue lymphatics, which our study identified as a significant predictor of delayed surgical site infection, although only in univariate analysis [26]. Therefore, structural rather than biological changes in the tissue microenvironment may be critical contributors to the risk of delayed surgical site infection.

The retrospective nature of this study introduces inherent limitations, potentially accounting for the lack of significance in several patient-specific risk factors. In addition, certain surgical factors during the perioperative period, including intraoperative bleeding, postoperative drainage, and drainage time, were omitted from the evaluation. Consequently, the potential impacts of these perioperative conditions on surgical site infection could not be taken into account. A comprehensive infectious disease work-up, including complete blood count, C-reactive protein, erythrocyte sedimentation rate, and microbial culture, was not available due to logistical constraints in real-world practice. Of note, the special circumstances of this study based on Asian women with relatively smaller breast volumes cannot be underestimated. The generalizability of our results to the Western population with larger breast size remains to be validated [27]. Also, the presence of fluid in the primary tumor area can be influenced by postsurgical complications and the time interval between surgery and radiotherapy planning. For patients undergoing adjuvant chemotherapy, the sequencing of chemotherapy prior to radiotherapy may further alter the tumor bed characteristics. These factors, which were not fully accounted for in this study, could have influenced the volumetric analysis and should be considered in future research to refine risk stratification and treatment planning. Finally, the data are inconclusive regarding the impacts of a specific hypofractionation regimen on surgical site infection. Additional data accumulation with a specific focus on the effects of RT regimens is desirable.

## 5. Future Perspectives

Future studies should aim to address the gaps and limitations identified in this study to advance our understanding of surgical site infections in breast cancer patients undergoing breast-conserving therapy. Conducting a comparative analysis of infection rates between hypofractionated and conventionally fractionated RT to better understand the impact of RT regimens may be a good starting point. Investigating the impact of volumetric parameters, including the cavity-to-breast ratio, across RT techniques and patient demographics may also provide insights into targeted prevention strategies for surgical site infections. These insights may allow for the optimal individualization of not only infection prophylaxis but also RT delivery. The predictive role of perioperative antibiotic prophylaxis in the occurrence of breast infections is another area of investigation.

## 6. Conclusions

This study highlights the importance of volumetric parameters, especially the cavity-to-breast ratio, as significant predictors of surgical site infection in a pure cohort of early breast cancer patients undergoing BCS and hypofractionated RT. Tailored approaches, including close follow-up, prophylactic aspiration, and prophylactic antibiotics, may be considered for high-risk patients with significant cavity-to-breast ratios. While these strategies have the potential to reduce the morbidity associated with surgical site infections and improve clinical outcomes, further studies are needed to confirm their effectiveness and impact on tumor control.

## Figures and Tables

**Table 1:** Patient, tumor, and treatment characteristics.

	N = 145 (%)
Age (years)	median 54 (range: 26–82)
BMI (kg/m^2^)	median 24.2 (range: 17.7–38.3)
Smoking history	
None	130 (89.7%)
Old	10 (6.9%)
Current	5 (3.4%)
DM	
Yes	17 (11.7%)
No	128 (88.3%)
HTN	
Yes	43 (29.7%)
No	102 (70.3%)
Axillary surgery	
SLNB	125 (86.2%)
ALND	9 (6.2%)
None	11 (7.6%)
T stage	
Tis	31 (21.4%)
T1mi	1 (0.7%)
T1a	8 (5.5%)
T1b	19 (16.1%)
T1c	54 (37.2%)
T2	32 (22.1%)
N stage	
N0	124 (85.5%)
N1a	10 (6.9%)
Nx	11 (7.6%)
ER status	
Negative	20 (13.8%)
Positive	125 (86.2%)
PR status	
Negative	29 (20%)
Positive	116 (80%)
Her2 status	
Negative	121 (83.5%)
Positive	24 (16.5%)
Ki-67 (%)	median 12 (range: 1–95)
Adjuvant chemotherapy	
Yes	73 (50.3%)
None	72 (49.7%)
Adjuvant hormonal therapy	
Tamoxifen	58 (40%)
Aromatase inhibitor	66 (45.5%)
None	21 (14.5%)
Adjuvant targeted therapy	
Yes	14 (9.7%)
None	131 (90.3%)
Radiation dose	
4005 cGy	88 (60.7%)
4256 cGy	57 (39.3%)
Boost	
Yes	132 (91%)
No	13 (9%)

Abbreviations: BMI: body mass index; DM: diabetes mellitus; HTN: hypertension; SLNB: sentinel lymph node biopsy; ALND: axillary lymph node dissection; ER: estrogen receptor; PR: progesterone receptor; Her2: human epidermal growth factor receptor 2.

**Table 2:** Rates of surgical site infection and acute toxicity after hypofractionated radiotherapy.

Toxicity	N = 145 (%)	Grade 1	Grade 2	Grade 3
Surgical site infection	7 (4.8)	2 (1.4)	1 (0.7)	4 (2.8)
Dermatitis	143 (98.6)	140 (96.6)	3 (2.1)	0 (0.0)
Breast pain	37 (25.5)	34 (23.4)	3 (2.1)	0 (0.0)
Breast edema	55 (37.9)	54 (37.2)	1 (0.7)	0 (0.0)
Arm edema	1 (0.7)	1 (0.7)	0 (0.0)	0 (0.0)

**Table 3:** Volumetric parameters of breast surgery and whole breast radiotherapy as potential risk factors for surgical site infection.

Volumetric Parameters	Median (Range)
Tumor size (cm)	1.4 (0.1–5.0)
Specimen width (cm)	7.7 (2.7–18.0)
Specimen depth (cm)	6.0 (2.2–17.5)
Specimen height (cm)	3.0 (0.8–6)
Specimen volume (cc)	144.0 (9.2–1417.5)
Skin width (cm)	6.0 (1.5–16.0)
Skin depth (cm)	1.7 (0.7–5.4)
Breast volume (cc)	680.9 (191.9–1485.8)
Surgical cavity volume (cc)	13.1 (0–216)
Cavity-to-breast ratio (%)	1.78 (0–16.8)

**Table 4:** Logistic regression analysis of risk factors for surgical site infection.

Risk Factors	Univariate Analysis *	*p*	Multivariate Analysis **	*p*
	OR (95% CI)		OR (95% CI)	
Tumor size	2.01 (1.07–3.74)	0.025	1.74 (0.86–3.46)	0.108
Specimen volume	1.00 (0.99–1.01)	0.076		
Surgical cavity volume ***	1.03 (1.01–1.07)	0.013		
Breast volume ***	1.00 (0.99–1.01)	0.073		
Cavity-to-breast ratio ***	1.29 (1.08–1.56)	0.005	1.24 (1.01–1.54)	0.039
BMI	1.23 (1.04–1.46)	0.014	1.18 (0.98–1.42)	0.071
RT regimen	4.13 (0.86–29.60)	0.097		
(4005 cGy vs. 4256 cGy)				

Abbreviations: OR: odds ratio; CI: confidence interval; BMI: body mass index; RT: radiation therapy. * Univariate analysis shows only the factors with *p* < 0.10. ** Multivariate analysis includes the factors significant at *p* < 0.05 in the univariate analysis. *** Among interrelated factors, the factor with the highest significance in the univariate analysis was included in the multivariate analysis to avoid a confounding effect.

## Data Availability

The datasets generated and/or analyzed during the current study are available from the corresponding author upon reasonable request.

## References

[1] Kim K.S., Shin K.H., Choi N., Lee S.W. (2016). Hypofractionated whole breast irradiation: New standard in early breast cancer after breast-conserving surgery. Radiat. Oncol. J..

[2] Yarnold J., Ashton A., Bliss J., Homewood J., Harper C., Hanson J., Haviland J., Bentzen S., Owen R. (2005). Fractionation sensitivity and dose response of late adverse effects in the breast after radiotherapy for early breast cancer: Long-term results of a randomised trial. Radiother. Oncol..

[3] Owen J.R., Ashton A., Bliss J.M., Homewood J., Harper C., Hanson J., Haviland J., Bentzen S.M., Yarnold J.R. (2006). Effect of radiotherapy fraction size on tumour control in patients with early-stage breast cancer after local tumour excision: Long-term results of a randomised trial. Lancet Oncol..

[4] Group S.T., Bentzen S.M., Agrawal R.K., Aird E.G., Barrett J.M., Barrett-Lee P.J., Bliss J.M., Brown J., Dewar J.A., Dobbs H.J. (2008). The UK Standardisation of Breast Radiotherapy (START) Trial A of radiotherapy hypofractionation for treatment of early breast cancer: A randomised trial. Lancet Oncol..

[5] Group S.T., Bentzen S.M., Agrawal R.K., Aird E.G., Barrett J.M., Barrett-Lee P.J., Bentzen S.M., Bliss J.M., Brown J., Dewar J.A. (2008). The UK Standardisation of Breast Radiotherapy (START) Trial B of radiotherapy hypofractionation for treatment of early breast cancer: A randomised trial. Lancet.

[6] Haviland J.S., Owen J.R., Dewar J.A., Agrawal R.K., Barrett J., Barrett-Lee P.J., Dobbs H.J., Hopwood P., Lawton P.A., Magee B.J. (2013). The UK Standardisation of Breast Radiotherapy (START) trials of radiotherapy hypofractionation for treatment of early breast cancer: 10-year follow-up results of two randomised controlled trials. Lancet Oncol..

[7] Whelan T.J., Pignol J.P., Levine M.N., Julian J.A., MacKenzie R., Parpia S., Shelley W., Grimard L., Bowen J., Lukka H. (2010). Long-term results of hypofractionated radiation therapy for breast cancer. N. Engl. J. Med..

[8] Indelicato D.J., Grobmyer S.R., Newlin H., Morris C.G., Haigh L.S., Copeland E.M., Mendenhall N.P. (2006). Delayed breast cellulitis: An evolving complication of breast conservation. Int. J. Radiat. Oncol. Biol. Phys..

[9] Degnim A.C., Throckmorton A.D., Boostrom S.Y., Boughey J.C., Holifield A., Baddour L.M., Hoskin T.L. (2012). Surgical site infection after breast surgery: Impact of 2010 CDC reporting guidelines. Ann. Surg. Oncol..

[10] O’Connor R.Í., Kiely P.A., Dunne C.P. (2020). The relationship between post-surgery infection and breast cancer recurrence. J. Hosp. Infect..

[11] Xue D.Q., Qian C., Yang L., Wang X.F. (2012). Risk factors for surgical site infections after breast surgery: A systematic review and meta-analysis. Eur. J. Surg. Oncol..

[12] Exarchos G., Metaxa L., Constantinidou A., Kontos M. (2019). Delayed Breast Cellulitis following Surgery for Breast Cancer: A Literature Review. Breast Care.

[13] (2017). AJCC Cancer Staging Manual.

[14] NCCN Clinical Practice Guidelines in Oncology: Breast Cancer. https://www.nccn.org/professionals/physician_gls/pdf/breast.pdf.

[15] Breast Cancer Atlas for Radiation Therapy Planning: RTOG Consensus Definitions. https://www.srobf.cz/downloads/cilove-objemy/breastcanceratlas.pdf.

[16] Common Terminology Criteria for Adverse Events (CTCAE). https://ctep.cancer.gov/protocoldevelopment/electronic_applications/docs/ctcae_v5_quick_reference_5x7.pdf.

[17] Olsen M.A., Chu-Ongsakul S., Brandt K.E., Dietz J.R., Mayfield J., Fraser V.J. (2008). Hospital-associated costs due to surgical site infection after breast surgery. Arch. Surg..

[18] Meattini I., Becherini C., Boersma L., Kaidar-Person O., Marta G.N., Montero A., Offersen B.V., Aznar M.C., Belka C., Brunt A.M. (2022). European Society for Radiotherapy and Oncology Advisory Committee in Radiation Oncology Practice consensus recommendations on patient selection and dose and fractionation for external beam radiotherapy in early breast cancer. Lancet Oncol..

[19] Shaitelman S.F., Schlembach P.J., Arzu I., Ballo M., Bloom E.S., Buchholz D., Chronowski G.M., Dvorak T., Grade E., Hoffman K.E. (2015). Acute and Short-term Toxic Effects of Conventionally Fractionated vs Hypofractionated Whole-Breast Irradiation: A Randomized Clinical Trial. JAMA Oncol..

[20] Srivastava V., Basu S., Shukla V.K. (2012). Seroma formation after breast cancer surgery: What we have learned in the last two decades. J. Breast Cancer.

[21] Adwall L., Hultin H., Mani M., Norlen O. (2022). Prospective Evaluation of Complications and Associated Risk Factors in Breast Cancer Surgery. J. Oncol..

[22] Nicolas P., Yazdan Y., Marie-Pierre C., Stéphanie C., Sylvia G., Jean-Charles N., Danièle L., Charles F., Jacques B. (2007). Prevention of surgical site infection after breast cancer surgery by targeted prophylaxis antibiotic in patients at high risk of surgical site infection. J. Surg. Oncol..

[23] Vilar-Compte D., de Iturbe I.Á., Martín-Onraet A., Pérez-Amador M., Sánchez-Hernández C., Volkow P. (2008). Hyperglycemia as a risk factor for surgical site infections in patients undergoing mastectomy. Am. J. Infect. Control.

[24] Chung C.U., Wink J.D., Nelson J.A., Fischer J.P., Serletti J.M., Kanchwala S.K. (2015). Surgical Site Infections after Free Flap Breast Reconstruction: An Analysis of 2899 Patients from the ACS-NSQIP Datasets. J. Reconstr. Microsurg..

[25] Jacobson L.K., Johnson M.B., Dedhia R.D., Niknam-Bienia S., Wong A.K. (2017). Impaired wound healing after radiation therapy: A systematic review of pathogenesis and treatment. JPRAS Open.

[26] Kataru R.P., Park H.J., Baik J.E., Li C., Shin J., Mehrara B.J. (2020). Regulation of Lymphatic Function in Obesity. Front. Physiol..

[27] Bhoo-Pathy N., Yip C.H., Hartman M., Uiterwaal C.S., Devi B.C., Peeters P.H., Taib N.A., van Gils C.H., Verkooijen H.M. (2013). Breast cancer research in Asia: Adopt or adapt Western knowledge?. Eur. J. Cancer.

